# Supplementary material for: Epidemiological and phylogenetic analyses of public SARS-CoV-2 data from Malawi
Source: PLOS Glob Public Health. 2025 Mar 21;5(3):e0003943. doi: 10.1371/journal.pgph.0003943 (PMC11927878; doi:10.1371/journal.pgph.0003943)
Supplement: S1 Text — (PDF) [file pgph.0003943.s001.pdf]

Supplementary material for the Epidemiological and phylogenetic analyses of public SARS-CoV-2 data from Malawi

1 Generalised Additive Models (GAM)

Implemented in R using the mgcv package, generalised additive models with the negative binomial family were fitted to data to describe trends of cases and deaths over time. Two fixed covariates, day and day of week were considered and the corresponding model smooth term of class s was defined for the day explanatory variable; with a cyclic cubic regression spline (bs = "cc") and enough basis dimension ($k = 45$). Below is the model,

$$g(\mu_i) = \mathbf{A_i}\theta + f_1(x_{1i}) + f_2(x_{2i})$$

where $\mu_i \equiv \mathbb{E}(Y_i)$ and $Y_i \sim \mathbf{EF}(\mu_\mathbf{i}, \phi)$ is a response variable (cases or deaths) with an exponential family distribution (negative binomial) with mean μ_i and scale parameter ϕ. $\mathbf{A_i}$ is the row of a model matrix, θ is the parameter vector, f_1 and f_2 are smooth functions of covariates, x_1 and x_2. A smooth function is denoted by;

$$f(x) = \sum_{j=1}^{k} b_j(x)\beta_j$$

where β_j represents the value of unknown parameter and b_j is the basis of expansion (i.e x^0, x^1, x^2, x^3 e.t.c) [1].

Negative binomial and quasi-poisson distributions are widely used in epidemiology for overdispersed count variables where variance is greater than the mean [1]. Negative binomial assumes that variance is a quadratic function of the mean while quasi-poisson model assumes that variance is a linear function of the mean. In this study, the variance to mean ratio of both case and death data was high, 435.5 and 15.2 respectively, implying that data was overdispersed therefore, negative binomial or quasipoisson family distribution was ideal. However, negative binomial family distribution was chosen because research shows that it performs way better with real-world data than the others because they give a close fit, especially for data that contains $< 30\%$ of significant zero counts [2].

2 Growth rate and Doubling time of SARS-COV-2

Occurrence of new cases in a period, the incidence of a disease is denoted by;

$$y(t) = y_0 \mathrm{e}^{rt} + \text{noise}, \quad (1)$$

where r is growth rate and y_0 is the disease incident at time 0. Number of cases or deaths at time t, $y(t)$ is proportional to $\mathrm{e}^{s(t)}$;

$$y(t) \propto \mathrm{e}^{s(t)}$$

where $s(t)$ are smooth functions. The time derivative of the smooth functions returns the instantaneous growth rate, $r = \dot{s}(t) = \dot{f}_1(x_1) + \dot{f}_2(x_2)$ where x_1 and x_2 are day and day-of-week, respectively [3] [4].

$$T_D = \frac{\ln(2)}{\dot{s}(t)} \tag{2}$$

References

1. Wood SN. Generalized additive models: an introduction with R. Chapman and Hall/CRC; 2006.

2. Kasyoki Muoka A, Owino Ngesa O, Gichuhi Waititu A. Statistical models for count data. 2016;.

3. Anzai A, Nishiura H. Doubling time of infectious diseases. Journal of Theoretical Biology. 2022;554:111278.

4. Number R. and Growth Rate (r) of the COVID-19 Epidemic in the UK: Methods of Estimation, Data Sources, Causes of Heterogeneity, and Use as a Guide in Policy Formulation. The Royal Society: London, UK. 2020; p. 1–86.
