# Supplementary material for: Epidemiological and phylogenetic analyses of public SARS-CoV-2 data from Malawi
Source: PLOS Glob Public Health. 2025 Mar 21;5(3):e0003943. doi: 10.1371/journal.pgph.0003943 (PMC11927878; doi:10.1371/journal.pgph.0003943)
Supplement: S1 Fig — (PDF) [file pgph.0003943.s002.pdf]

Supplementary material for the Epidemiological and phylogenetic analyses of public SARS-CoV-2 data from Malawi

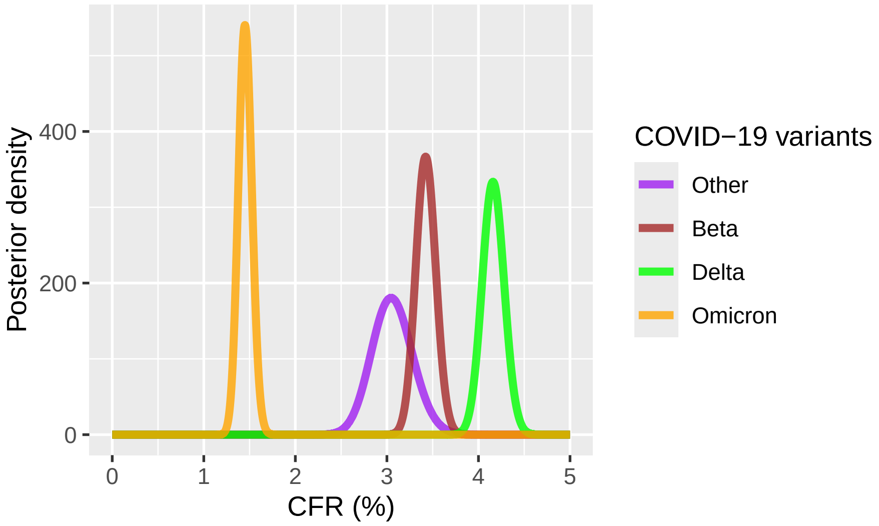

Posterior distribution of CFR for the variants: Omicron has the least CFR while Delta has the highest CFR.the distributions for Other and Beta variants almost overlap.
