# Supplementary material for: Epidemiological and phylogenetic analyses of public SARS-CoV-2 data from Malawi
Source: PLOS Glob Public Health. 2025 Mar 21;5(3):e0003943. doi: 10.1371/journal.pgph.0003943 (PMC11927878; doi:10.1371/journal.pgph.0003943)
Supplement: S3 Fig — (PDF) [file pgph.0003943.s004.pdf]

Supplementary material for the Epidemiological and phylogenetic analyses of public SARS-CoV-2 data from Malawi

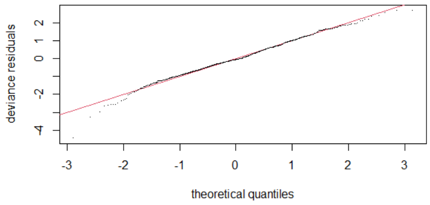

(a) Q-Q plot

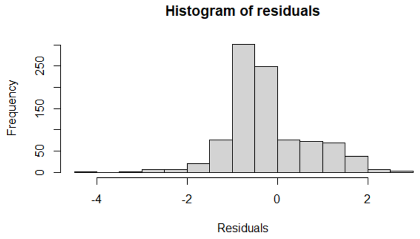

(b) Histogram

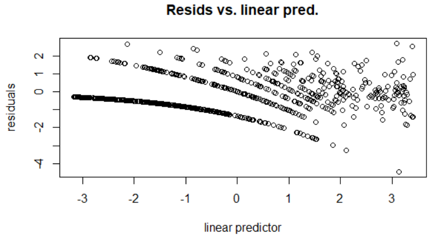

(c) Residuals against Linear Predictor

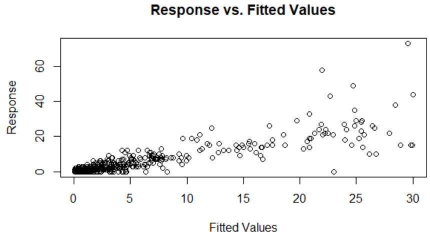

(d) Response versus Fitted values

Model diagnostic plots for deaths
