# Supplementary material for: Epidemiological and phylogenetic analyses of public SARS-CoV-2 data from Malawi
Source: PLOS Glob Public Health. 2025 Mar 21;5(3):e0003943. doi: 10.1371/journal.pgph.0003943 (PMC11927878; doi:10.1371/journal.pgph.0003943)
Supplement: S1 Table — (PDF) [file pgph.0003943.s007.pdf]

Supplementary material for the Epidemiological and phylogenetic analyses of public SARS-CoV-2 data from Malawi

SARS-Cov-2 lineages grouped into variants and named according to names given by the World Health Organisation (WHO).

COVID-19 variant				
Alpha (6 sequences)	Beta (492 sequences)	Delta (569 sequences)	Omicron (273 sequences)	Other (82 sequences)
B.1.1.7	B.1.351	AY.104	BA.1	A.23.1
	B.1.36	AY.116	BA.1.1	B
		AY.122	BA.1.13	B.1
		AY.30	BA.1.14	B.1.1
		AY.37	BA.1.14.2	B.1.1.1
		AY.38	BA.1.17	B.1.1.33
		AY.45	BA.1.17.2	B.1.1.375
		AY.46	BA.1.18	B.1.1.412
		AY.6	BA.1.19	B.1.1.448
		B.1.617.2	BA.1.21	B.1.1.54
			BA.1.9	B.1.177
			BA.2	B.6
			BA.2.65	
			BA.4	
			BA.4.1	
			BA.4.1.9	
			BA.4.6.1	
			BA.4.7	
			BA.5	
			BA.5.2	
			BQ.1.1	

The collected sequences in fasta format were aligned to identify regions of similarities. The sequence alignment process used MAFFT, a multiple alignment program for amino acid or nucleotide sequences, which we coded in python-jupyter notebook. The aligned sequences together with the reference sequence of Wuhan city, China, were later processed into evolution trees using IQ-TREE, a stochastic algorithm that builds phylogenetic trees by the maximum likelihood method [1]. IQ-TREE, which is an efficient, open-source software for phylogenomic inference was chosen for its efficiency in selecting the best model for tree reconstruction. The tree was rooted with a reference sequence of Wuhan using a model $GTR + F + I + I + R5$ that emerged as the best model through the model finder [2]. TreeTime version 0.82 [3] was used to visualise the reconstructed national phylogenetic tree on a time scale while the trees

for individual variants were visualised and coloured by region using an online tool for displaying, annotating and managing trees called an Interactive Tree Of Life (iTOL).

References

1. Minh BQ, Schmidt HA, Chernomor O, Schrempf D, Woodhams MD, Von Haeseler A, et al. IQ-TREE 2: new models and efficient methods for phylogenetic inference in the genomic era. Molecular biology and evolution. 2020;37(5):1530–1534.

2. Kalyaanamoorthy S, Minh BQ, Wong TK, Von Haeseler A, Jermiin LS. ModelFinder: fast model selection for accurate phylogenetic estimates. Nature methods. 2017;14(6):587–589.

3. Sagulenko P, Puller V, Neher RA. TreeTime: Maximum-likelihood phylodynamic analysis. Virus evolution. 2018;4(1):vex042.
