# Supplementary material for: Epidemiological and phylogenetic analyses of public SARS-CoV-2 data from Malawi
Source: PLOS Glob Public Health. 2025 Mar 21;5(3):e0003943. doi: 10.1371/journal.pgph.0003943 (PMC11927878; doi:10.1371/journal.pgph.0003943)
Supplement: S2 Table — (PDF) [file pgph.0003943.s008.pdf]

Supplementary material for the Epidemiological and phylogenetic analyses of public SARS-CoV-2 data from Malawi

Estimation results for the GAM model-cases, the intercept is significant.

	Estimate	Standard Error	$z-$value	$p-$value	CI
Intercept	3.070814	0.060623	50.654	$< 2 \times 10^{-16}$	$(3.01, 3.13)$
DayOfWeek	-0.001999	0.013506	-0.148	0.882	$(-0.02, 0.01)$
