# Supplementary material for: Epidemiological and phylogenetic analyses of public SARS-CoV-2 data from Malawi
Source: PLOS Glob Public Health. 2025 Mar 21;5(3):e0003943. doi: 10.1371/journal.pgph.0003943 (PMC11927878; doi:10.1371/journal.pgph.0003943)
Supplement: S3 Table — (PDF) [file pgph.0003943.s009.pdf]

# Supplementary material for the Epidemiological and phylogenetic analyses of public SARS-CoV-2 data from Malawi

Distribution of SARS-Cov-2 variants across the regions

| Variant | Southern region | Central region | Northern region | No region | Total | Total (%) |
|---|---|---|---|---|---|---|
| Alpha | 4 | 0 | 0 | 2 | 6 | 0.42 |
| Beta | 180 | 78 | 36 | 198 | 492 | 34.6 |
| Delta | 383 | 42 | 5 | 139 | 569 | 40.01 |
| Omicron | 213 | 21 | 37 | 2 | 273 | 19.2 |
| Other | 25 | 3 | 2 | 45 | 82 | 5.77 |
