# Supplementary material for: Epidemiological and phylogenetic analyses of public SARS-CoV-2 data from Malawi
Source: PLOS Glob Public Health. 2025 Mar 21;5(3):e0003943. doi: 10.1371/journal.pgph.0003943 (PMC11927878; doi:10.1371/journal.pgph.0003943)
Supplement: S4 Table — (PDF) [file pgph.0003943.s010.pdf]

Supplementary material for the Epidemiological and phylogenetic analyses of public SARS-CoV-2 data from Malawi

Proportions and Confidence Interval of SARS-Cov-2 variants.
P is the proportion, CI is the 95% confidence interval and SE is the standard error.

Variant	Southern region			Central region			Northern region			No region		
	P	CI	SE	P	CI	SE	P	CI	SE	P	CI	SE
Alpha	0.67			0			0			0.33		
Beta	0.37	$0.32, 0.41$	0.05	0.16	$0.13, 0.20$	0.03	0.073	$0.05, 0.10$	0.02	0.40	$0.34, 0.45$	0.06
Delta	0.67	$0.63, 0.71$	0.04	0.07	$0.05, 0.10$	0.02	0.01	$0.00, 0.02$	0.01	0.24	$0.21, 0.28$	0.03
Omicron	0.78	$0.73, 0.83$	0.05	0.08	$0.05, 0.12$	0.03	0.14	$0.10, 0.18$	0.04	0.01	$0.00, 0.03$	0.01
Other	0.37	$0.26, 0.48$	0.11	0.04	$0.01, 0.11$	0.04	0.05	$0.02, 0.13$	0.04	0.55	$0.44, 0.66$	0.11
