# Supplementary material for: Epidemiological and phylogenetic analyses of public SARS-CoV-2 data from Malawi
Source: PLOS Glob Public Health. 2025 Mar 21;5(3):e0003943. doi: 10.1371/journal.pgph.0003943 (PMC11927878; doi:10.1371/journal.pgph.0003943)
Supplement: Attachment — Submitted filename: PGPH-D-24-01510_Reviewer Recommendation.pdf [file pgph.0003943.s014.pdf]

Manuscript Number PGPH-D-24-01510

Reviewer Recommendation and Comments for

ABSTRACT

<u>Considerer rephrasing</u>

- Consider adding a brief introductory sentence at the beginning to set the stage for the reader. For example, *"The COVID-19 pandemic has had varying impacts across different regions, necessitating localized data-driven responses."*

- Now: "present modelling analyses of SARS-CoV-2 epidemiology and phylogenetics in Malawi from 2 April 2020 to 19 October 2022" is clear, but could be shortened slightly for efficiency:
- Proposal: "present a modeling analysis of SARS-CoV-2 epidemiology and phylogenetics in Malawi between 2 April 2020 and 19 October 2022."

- The statement "Some sequences associated with the Alpha variant were present, but these did not appear to drive a major wave as they did in some other countries" is informative but might benefit from a brief explanation or hypothesis as to why Alpha did not drive a major wave in Malawi.

- The abstract ends with a strong argument about the potential for real-time analysis in Malawi and similar countries. However, it might benefit from a slight rephrasing for impact:
- We suggest that real-time analyses should be considered in Malawi and other, where similar computational and data resources are available."

- The repetition of "Omicron" in "Omicron BA.1; (5) Other Omicron" might be rephrased to avoid redundancy.
- Consider merging these points, e.g., "Omicron BA.1 and other sublineages."

MATERIALS AND METHODS

<u>Considerer rephrasing</u>

- "Malawi is a landlocked country that is bordered by Mozambique to the East, South and Southwest, Tanzania to the north and northeast and Zambia to the West and Northwest" could be streamlined for clarity.
- "Malawi is a landlocked country bordered by Mozambique to the east, south, and southwest; Tanzania to the north and northeast; and Zambia to the west and northwest."

- "The country is located at latitudes between 9◦ 22' S and 17◦ 03' S and longitudes between 33◦ 40' E and 35◦ 55' E".
- "Malawi lies between latitudes 9◦ 22' S and 17◦ 03' S, and longitudes 33◦ 40' E and 35◦ 55' E."

- "The country has three regions; North, Central and South; and 28 districts"
- "Malawi is divided into three regions—North, Central, and South—and comprises 28 districts."

- "In a year before the 2018 household census across the country, there were 6.3 all-cause deaths and 32.8 births per 1,000 persons"
- "In the year preceding the 2018 household census, the country recorded 6.3 all-cause deaths and 32.8 births per 1,000 persons."

- "Malawi is categorised as a Low-Income country [21] with a fragile health system [22]"
- "Malawi is categorized as a Low-Income country [21], with a health system that faces significant challenges, including limited financial and human resources [22]."

RESULTS

The overall structure of the "Results" section is logical; however, the readability can be improved by slightly rephrasing some sentences for clarity.

<u>Rephrasing some sentences for clarity</u>

- "The overall CFR for the whole study period is 3.05% (95% credible interval (2.94, 3.16)%)" is clear but could be more concise.
- "The overall CFR for the entire study period was 3.05% (95% CrI: 2.94%–3.16%)."

- "CFR = 4.16%, CrI = (3.93, 4.40)"
- "CFR: 4.16% (95% CrI: 3.93%–4.40%)."

- "Delta was more severe (CFR = 4.16%, CrI = (3.93, 4.40)) than the other variants,"
- the word "severe" might be replaced with "lethal" or "had a higher fatality rate," as "severe" might imply clinical severity rather than mortality specifically.

- Case Fatality Rate for SARS-Cov-2 variants"
- "Case Fatality Rates for SARS-CoV-2 Variants Observed in Malawi."

- "The full posterior distribution of CFR for the variants is shown in Supplementary Material S1, showing the CFR of Other and Beta overlapping,"
- "The full posterior distribution of CFRs, which indicates overlapping CFRs for the 'Other' and Beta variants, is provided in Supplementary Material S1."

- "The posterior distribution of CFR for the variants suggests the same, that Delta has the highest CFR and Omicron has the lowest"
- "The posterior distribution of CFRs further confirms that the Delta variant had the highest fatality rate, while the Omicron variant had the lowest."

- "Five peaks are clearly visible in the plot, representing the five waves however, the last peak is very short" could be rephrased for clarity.
- "Five distinct peaks are visible in the plot, representing five waves of infection, though the final peak is notably brief."

<u>Numerical Data Presentation:</u>

- Ensure consistent formatting of numerical data throughout the text. For example, "88, 064" should be "88,064" (no space after the comma), and "1, 316" should be "1,316."
- When mentioning specific numbers like "95 new reported cases every day" and "variance was 41,200," consider adding brief context or comparisons to help readers understand the significance of these figures.

- The explanation of the GAM model and its significance is good but could be made clearer. For example: "A Generalized Additive Model (GAM) was fit to the case data using a negative binomial distribution with a log link function. This model accounted for day and day-of-week effects and is visualized in Fig 2C."

- Ensure consistency in how variants are referred to throughout the text. For instance, "Omicron," "Beta," "Delta," and "Other" should always be capitalized and referred to consistently (e.g., "Other" variant instead of "non-VOC").

- "Malawi's first variant was non-VOC, which emerged before Alpha (Other)" is somewhat unclear.
- "The first variant identified in Malawi was non-VOC, which predated the Alpha variant and is referred to here as 'Other.'"

DISCUSSION

<u>Rephrasing some sentences for clarity</u>

- "The far-reaching consequences of COVID-19 throughout the world as well as individual states motivates the need for extensive research around the pandemic,"
- "The global and local impacts of COVID-19 have driven the need for extensive research on the pandemic."

- "It was, therefore, worth understanding the disease dynamics, the evolution of the virus and the burden the pandemic posed on a low-income country like Malawi to aid in the formation of policies and interventions at present or in the future"
- "Understanding the disease dynamics, viral evolution, and pandemic burden in a low-income country like Malawi is crucial for informing current and future policy interventions."

Correct, the sentence contains a typo

Line 317.

- “With the use of generalised additive models, **te infection’s trend and groethrate** were described while the use of IQtree, TreeTime and iTOL allowed the capturing of the times of the most recent common ancestors for the variants as well as the geographical distribution of the virus”.
- This should be corrected to "**the infection’s trend and growth rate** "

Rephrasing some sentences for clarity

- "Results show that Malawi encountered five waves of SARS-CoV-2 (see Fig 2A), and that each wave was dominated by one variant as the time tree, as Fig 5 illustrates,"
- "Results indicate that Malawi experienced five waves of SARS-CoV-2 (see Fig 2A), each dominated by a distinct variant, as illustrated by the time tree in Fig 5."

- "This chronological order agrees with the literature particularly study results of Anscombe (2023)"
- "This chronological order is consistent with the findings of Anscombe (2023) [43], who also observed a similar sequence of variant emergence in Malawi."

- "Comparing the waves in terms of cases, the study found that the first wave, Other was the least infectious of all" could be clarified.”
- "In comparing the waves by case numbers, the study found that the first wave, dominated by the 'Other' variant, was the least infectious, potentially due to low testing or under-reporting during the early pandemic phase."

- The $\chi 2$ test showed that proportions of COVID-19 variants across the regions of Malawi were significantly different implying that the variants posed unequal impact on the regions. Precisely, Omicron was worse in the southern and northern regions compared to the central region. Beta did not affect the southern region much, as Delta did in the north.
- The distribution of COVID-19 variants across the regions of Malawi varied significantly, indicating that the impact of the pandemic was not uniform across the country. Specifically, the Omicron variant had a more severe impact in the southern and northern regions compared to the central region. In contrast, the Beta variant had a relatively limited effect on the southern region, while the Delta variant had a more pronounced impact in the northern region.

Interpretation of Results:

- The interpretation of the impact of vaccination and variant infectiousness is clear but could be expanded slightly for depth.
- Proposal: "The reduced infectiousness of the Delta variant, coinciding with the vaccination rollout, suggests a potential impact of vaccination in slowing transmission and establishing herd immunity."

- The statement "Omicron was more aggressive despite that a good proportion of the population had been vaccinated" should be clarified to distinguish between "aggressiveness" in terms of transmission versus severity:
- Proposal: "Omicron was more transmissible despite a substantial portion of the population being vaccinated, though it resulted in fewer deaths compared to Beta and Delta."

CONCLUSIONS

The conclusion effectively recaps the main findings, but it would be better with a more organized synthesis. You could consider grouping related findings together. For example:

1) Summarize the variant distribution

2) Discuss the impact on case fatality rates

3) Conclude with recommendations for future focus areas.

For example:

- "The study identified five major waves of SARS-CoV-2 in Malawi, each dominated by different variants, with Delta and Beta being the most prevalent. While the overall case fatality rate was lower than in high-income countries, it was higher than in other low-income countries. The study also highlighted the critical periods of increased transmission and emphasized the need for targeted interventions during these times."
- "To mitigate future outbreaks, it is crucial to prioritize resource allocation to the northern and southern regions, which were most affected by Omicron. Additionally, ongoing surveillance and rapid response strategies should be emphasized, particularly during periods of high transmission."
- "Further research is needed to explore the role of pre-existing immunity, genetic factors, and the impact of early preventive measures on disease outcomes in Malawi."
- "Given the rapid genetic evolution of SARS-CoV-2, continued vigilance and adaptability in public health strategies are essential to manage future variants and pandemics."

Complete the reference.

- Now: of Malawi PHI. COVID-19 Surveillance Dashboard; 2022.
- Correct: The Public Health Institute of Malawi PHI. COVID-19 Surveillance Dashboard; 2022.
