# Supplementary material for: Epidemiological and phylogenetic analyses of public SARS-CoV-2 data from Malawi
Source: PLOS Glob Public Health. 2025 Mar 21;5(3):e0003943. doi: 10.1371/journal.pgph.0003943 (PMC11927878; doi:10.1371/journal.pgph.0003943)
Supplement: Attachment — Submitted filename: Response to reviewers_Epidemiologicalrevised2.pdf [file pgph.0003943.s015.pdf]

RESPONSE TO REVIEWS

Reviewer #1
Background

1. To be succinct and concise, it would help to remove the section on basic information on COVID-19 up to emergence in Wuhan (line 11). Perhaps just start with the situation as it affected Africa as a continent?
We removed the basic information on COVID-19 and revised the introduction section to focus specifically on COVID-19 in Africa. The introduction now begins as follows on the lines 2-4:
"Africa was the last continent to be hit by COVID-19, and the first case was reported on 14 February 2020 in Egypt. The virus spread across the continent within three months. Lesotho ......"

2. Similarly, consider condensing the (very nice but lengthy) discussion of the different variants to the issue at hand: the emergence in Malawi.
We rephrased the paragraph as follows on lines 6-14:
"According to the WHO, SARS-CoV-2 variants have one of six main names: Alpha; Beta; Gamma; Delta; Omicron; and Non Variants of concern (non-VOC), which throughout this study is referred to as `Other'. **(10).** Omicron was first identified in South Africa in November 2021, then later spread more rapidly to other countries. **(10).** Although Omicron is highly transmissible, disease severity is lower than for Delta. Delta emerged before Omicron and was first identified in India in late 2020. In late 2020 in South Africa, the Beta variant was discovered for the first time and it was believed to have many mutations, more severe and caused more hospitalisations and deaths than the other variants(10)"

3. "Malawi registered its first COVID-19 case on 2 April 2020 [13], two weeks after 39 declaring a state of national disaster on March 20, 2020" (lines 39-40): some readers may be confused as to why Malawi declared the state of emergency before a case was confirmed in its territory. Perhaps add "pre-emptively declaring"
We added the word "pre-emptively" on line 16

4. "A report by the International Food Policy Research Institute (IFPRI) of June 2020, described Malawi's response to the COVID-19 pandemic as quite satisfactory" (lines 48-9): 'quite satisfactory' sounds too informal.
The statement has been removed, together with the word "However" .

5. "This work is to our knowledge the first comprehensive analysis of the disease epidemiology and phylogenetics of COVID-19 in Malawi using public data." (lines 56-7): State your objectives more clearly, i.e., "We conducted, to our knowledge, the first comprehensive analysis..." As well, what were the sub-objectives for the epidemiological (case/death), phylogenetic, and geographical

analyses? How were you trying to link the sets of data to provide a more complete situational analysis?
Lines 33-39 of the revised copy provides the sub-objectives of the study.
The genomic data explains the time series as the sequences were used to identify the lineages that drove the major waves of the pandemic in. We used case/death data for the time series plots. Again, the phylogenetic analysis revealed spatial variability of the variants as such, we found it worth providing basic information of the distribution of cases and deaths in the country.

6. Similarly, a criticism of this kind of historical work is that it does not help with the actual management of the epidemic if not done in real-time. What would you say to that? How do you make sense of the overall picture of COVID-19 in Malawi with all these data used in tandem?
Having the methods and the R codes of this study's analysis ready, will help to analyse in real time a similar situation/pandemic in the future. In addition, the methods can be adopted, with some changes for a different disease or when a slightly different situation arises.

The overall picture of COVID-19 in Malawi is that five variants circulated in Malawi between April 2020 and January 2023, and all variants drove major waves except for Alpha. Case incidence was high in the cities, which could be due to higher testing capacity in the cities than in rural areas

7. Please remove this, as it isn't necessary: "The paper has been arranged in a way that the next section describes the data as well as the methods applied to the analyses. After the methods section, results from the analyses are presented and later discussed in the section that follows. The last section concludes the paper by providing the implications of the findings." (lines 64-7)
The paragraph was removed.

<u>Methods</u>

1. Under Population and study setting, it would help to compare some of the data with the Southern Africa region, i.e., how does urban/rural proportion and all cause deaths and births compare regionally, or with neighboring countries?
We added a paragraph , lines 57-66.
"As of 26 September 2024, the median age for Malawi, Zambia and Zimbabwe was 18 while the median age for Tanzania and Mozambique was 17 and 16, respectively. The proportion of urban population in Malawi (19%) is much lower than in Zambia (46%), Mozambique (41%), Tanzania (39%) or Zimbabwe (38%). In the year 2022, the crude birth rate per 1,000 people for Malawi (33) is low compared to that of Zambia (34), Tanzania (36) and Mozambique (36), but higher than that of Zimbabwe (30). All-cause death rate per 1,000 people is 7 for both Malawi and Zambia, which is lower than that of Zimbabwe (9), and Mozambique (9). However, the death rate of 7 per 1,000

people for Malawi is slightly higher than that of Tanzania, which is 6 per 1,000 people."

2. Under Data sources, please list the exact data sources used for cases and genetic information, i.e., the Malawi Ministry of Health COVID-19 database (just an example). Please remove this statement, which doesn't add much: "Publicly available datasets on cases, deaths and genome of SARS-CoV-2 were used in this study. This means that the analysis should be completely reproducible by all researchers with Internet access and a modern desktop or laptop personal computer."
We rephrased "This was extracted from Our World in Data (OWID) website" to "This was extracted from Our World in Data (OWID) COVID-19 database for daily confirmed cases and confirmed deaths" on lines 73-75.

The source for genomic data was rephrased to "COVID-19 sequence data for Malawi was accessed from an open database, GISAID (the Global Initiative 96 on Sharing All Influenza Data) [25–27], under EpiCoV". See lines 77-78

Then the introductory part under Data Sources was removed, as proposed.

3. "In this study, we use data on daily new cases of SARS-CoV-2 beginning from 2 April 2020 to 19 October 2022, and new deaths beginning from 7 April 2020 to 19 October 2022. This was extracted from the Our World in Data (OWID) website [24], and corresponds to a study period of about two and a half years.": Are these suspect cases or confirmed cases? Please be specific here are everywhere in the manuscript when referring to suspect/confirmed cases and/or deaths. I assume the reason for censoring at 19 October 2022 was this was the end date of this particular analysis, or was there another reason to do so? Also does the OWID actually come from the Ministry of Health? Please acknowledge this as accurately as possible.
The cases and deaths are confirmed, and not suspected. We added the word "confirmed" where appropriate. We added on line 73; "The day 19 October was the end date for this study"

4. Is FASTA an acronym? This is a general readership journal, so please spell out all acronyms at first use.
Yes, FASTA stands for "FAST-All", so the sentence was rephrased: "We downloaded the FASTA file containing the sequences" to "We downloaded the file containing the sequences in FAST-All (FASTA) format". See lines 80-81.

5. How representative were the sequences available?
We used all sequences collected from GISAID, and all variants under study were available

6. Can you give the reader more information about how these genetic sequences are derived? Who did the genetic analysis, what methods were used etc. I imagine this is fairly generic, but we would like to know some specifics.

We revised as follows:

"Multiple sequence alignment was performed on 1,436 sequences using a program known as multiple alignment using fast Fourier transform (MAFFT) [37], which was implemented in Python. The aligned sequences were later reconstructed into a tree using IQ-TREE [38] software package, which is a stochastic algorithm that builds trees by maximum likelihood methods. The tree was later fit to a calendar and visualised using TreeTime version 0.82 [35]. Sequences in a FASTA file were also categorised according to names that WHO and researchers agreed to give to specific COVID-19 lineages [36]. Five categories were formed as shown in a table in the Supplementary material S1. Alignment and tree reconstruction were performed before the trees were visualised in interactive tree of life (iTOL)". See lines 122-131

7. "We fitted case and death data using R statistical package, particularly Generalised Additive Models (GAMs) with log link function": Should say "R statistical software" and the GAM R package? (is that what you meant?) (lines 115-6)

We corrected this as that is what we meant.

8. I am happy that you looked at growth rate and doubling time, but can you explain to the reader why it is important to look at these quantities in an epidemiological analysis?

To understand and control infection's distribution, the knowledge of growth rate and doubling time plays a vital role in providing helpful planning tool. The following information was added to the section of growth rate and doubling time, see lines 106-108:

"This provides the authorities with information on the most infectious lineages, for planning of the infection's preventive and control measures"

9. Line 118: What is meant by the smoothed estimate of the logarithm of a mean signal?

We meant that if integer data is modelled as Poisson / Quasi-Poisson, it needs a positive real mean, and the log of that mean can take any real value.

10. "Day-of-week was considered because reported cases or deaths were seemingly low during weekends.": Can you explain in plain language why day-of-week effects are important to this analysis?

On lines 111-114, this was rephrased to;

"Day-of-week was considered because during weekends hospitals only accept and treat emergencies, implying that the number of people who report illness to the hospital during weekends is likely to be less than the number reporting illness during weekdays. This affects the figures of the reported cases and deaths during weekends."

11. Lines 144-6: I think you can remove this to be more concise: "This analysis would be helpful in the future when similar risks arise since provides scientifically proven information to the authorities

on whether or not the regions are equally affected, a useful factor to consider when allocating resources to the affected regions."
We removed the sentence

Results

1. First, give a recap on how many suspect and confirmed cases and sequences were included in the analysis.
The case and death data used in this research are all reported/confirmed cases. We added the following on lines 140-142:
"The study used 88,084 confirmed cases and 2,683 confirmed deaths for the epidemiological analysis, and the geographic data had a total of 86,035 confirmed cases. Genomic data comprised of 1,436 sequences."

2. Add "CrI = credible interval" to Table 1
We added "CrI = credible interval" in the last column of table 1.

3. "The variance was 41, 200 and 95% of the values for daily new cases were below 547.": Is something wrong with this sentence?
We removed the sentence.

4. Figure 3 (panels A, B) needs a more plain language title (not "Derivative of spline arising from GAM"). Add doubling time to the 2nd y-axis in panels D and E. The second bumps in growth rate and doubling time for each variant are interesting. What do they mean?
Title for panels A and B of figure 3 were revised to "COVID-19 growth rate" and "COVID-19 death rate", respectively. The second y-axis was added to panels C and D.
We have also added the following to the discussion of limitations, see lines 359-364:
"Since the phenomena observed will be driven by complex non-linear disease dynamics, the unpredictable, intricate patterns and interactions that occur within a disease process over time can lead to disproportionately large effects so, in observational statistical work like this it is always hard to give a definitive explanation of all phenomena in e.g. the growth rate and doubling time curves. We hope to return to this in future work." We believe that this addresses interesting observation about the second bumps for each variant in the growth rate and doubling time curves.

5. Figure 4: since you are emphasizing the increased incidence in cities, can you mark the cities of Blantyre, Zomba, Lilongwe and Mzuzu on the maps? Also check the spelling of incidence.
We labelled the four cities on the map, and corrected the spelling of "incidence" in panel C of figure 4.

6. Since you are using confirmed cases only for the mapping, how much bias is there towards cities where access to confirmatory analysis were surely more available than the rest of this largely rural country? At the very least, this is a limitation.
We added under the discussion section (Lines 352-355) the following as a limitation;
"..... the study used only confirmed cases for mapping case incidence, which could lead to biased results since case ascertainment and testing capacity in the cities is likely to be higher than in the rural areas, considering that over 80% of people live in rural areas."

7. Given that the majority (57.3%) of sequences were collected from the Southern Region, it does not make sense to me to do analysis by region. Please remove these analyses and limit analysis to the whole dataset, or within each region (or justify their use).
We would like to keep this analysis as it further investigates the geographical heterogeneity of not only the infection in general but also each variant. Although more than half of the sequences were obtained from the southern region, there are interesting results about "Other" variant for instance, which was significantly less in the southern region.

Discussion

1. I would prefer in the first paragraph of the discussion to list a few of the major findings of the study, rather than restate the methodological steps.
We rephrased the first paragraph of the discussion section as below.
"The global and local impacts of COVID-19 have driven the need for extensive research on the pandemic. Many countries were affected across various sectors, such as health, social, and economic. The panic was especially intense in low- and middle-income countries, given their already limited resources [13]. Understanding disease dynamics, viral evolution, and pandemic burden in a low-income country like Malawi is crucial for informing current and future policy interventions. The study finds a case fatality rate of 3% (credible interval: 2.8% to 3.1%) as of October 19, 2022, with an early doubling time ranging from 4 to 7 days. Disease incidence was relatively high in Malawi's four main cities (see Fig 4), likely due to these cities being commercial hubs where most business activities take place. This high incidence may also be attributed to the higher levels of case ascertainment and testing capacity in urban compared to rural areas. The increased incidence on Likoma Island is likely influenced by the low population density. Major variants were identified, and the phylogenetic trees for individual variants showed close sequences appearing in different regions, indicating some inter-regional mobility despite travel restrictions."

2. "Disease incidence was relatively high in the four cities of Malawi (see Fig 4), most likely because they are commercial areas where the majority of businesses take place": Do you think this may also be because testing was more readily available, therefore increasing the numerator?
That is true, we added on lines 319-320;

“This high case incidence could also be attributed to the fact that the level of case ascertainment and testing capacity in the cities is higher than in rural areas”

3. There are many limitations to these kinds of analyses that rely on passively-reported data on infections and deaths, and they certainly impact what you can say with these analysis. One obvious one that comes to mind in the bias in availability of testing (probably more likely in major cities than rural areas?). What about ascertainment of deaths in health facilities and in the communities? There are several others. Please discuss what the limitations are, and how they impact your findings.

We added the following to the limitations (lines 355-358);
“Case incidence in various geographical locations greatly depends on the level of case ascertainment in respective health facilities, which is believed to differ between urban and rural hospitals.”

4. Please don’t add new results into the Discussion/Conclusion (i.e., names of lineages).
We removed the first two lines of the conclusion in the old version.

5. I find the Discussion to be rather dry, as you focus on restating the findings, with some comparisons with other studies that provide evidence to square the findings. I think what is missing is **how the three sets of findings and their limitations, provide a more comprehensive picture of the spatial and temporal situation of COVID-19 transmission and deaths in Malawi**. Similarly, is there anything that you found is surprising or contrasts with literature from Malawi and the Southern Africa region? I find this kind of discussion essential to this kind of analysis and strongly encourage the authors to expand on this in a few paragraphs.
The discussion now includes how the findings and the study limitations provide a broader picture of the spatiotemporal situation of COVID-19 in Malawi. Additionally, we added the following surprising findings on lines 344-351:
“Literature shows that the Omicron variant first emerged in South Africa and Botswana in November 2021. Surprisingly, this study finds that Omicron may have first appeared in Malawi as early as December 2020, although it took longer to mutate into multiple sub-lineages. The study also finds that the highest case incidences are observed in urban areas, primarily in cities. This contrasts with the findings of a spatiotemporal study of COVID-19 in Malawi, which used Geographic Information System (GIS) data and found a higher prevalence in Thyolo District than in the cities of Mzuzu and Zomba.”

6. The Conclusion, as it stands, is rather wordy. It should be limited to a main overall statement of the major findings.

We rephrased the conclusion to;
“We analyzed the cases, deaths, genomic, and geographical data of COVID-19 in Malawi, identifying five major waves, each dominated by different variants, with Delta and Beta being the most prevalent. While the overall case fatality rate was lower than in high-income countries, it was higher than in other low-income countries. The study also highlighted critical periods of increased transmission and emphasized the need for targeted interventions during these times. The early doubling time of infections was found to be higher than in other regions. The high case incidence observed in cities was likely due to several factors, including population density and testing capacity, among others; therefore, more studies considering these factors are required. Further research is also needed to explore the role of pre-existing immunity, genetic factors, and the impact of early preventive measures on disease outcomes in Malawi. To mitigate future outbreaks, it is crucial to prioritize resource allocation to the northern and southern regions, which were most affected by Omicron. Additionally, ongoing surveillance and rapid response strategies should be emphasized, particularly during periods of high transmission. Given the rapid genetic evolution of SARS-CoV-2, continued vigilance and adaptability in public health strategies are essential for managing future variants and pandemics.”

Reviewer #2

<u>Abstract</u>

Considerer rephrasing

1. Consider adding a brief introductory sentence at the beginning to set the stage for the reader. For example, "The COVID-19 pandemic has had varying impacts across different regions, necessitating localized data-driven responses."
   We added the phrase.

2. Now: "present modelling analyses of SARS-CoV-2 epidemiology and phylogenetics in Malawi from 2 April 2020 to 19 October 2022" is clear, but could be shortened slightly for efficiency:
   <u>Proposal:</u> "present a modeling analysis of SARS-CoV-2 epidemiology and phylogenetics in Malawi between 2 April 2020 and 19 October 2022."
   We accepted the proposal.

3. The statement "Some sequences associated with the Alpha variant were present, but these did not appear to drive a major wave as they did in some other countries" is informative but might benefit from a brief explanation or hypothesis as to **why Alpha did not drive a major wave in Malawi**.
   We cannot be certain but it could be due to the competition from Delta, which is more infectious than itself, since the few Alpha sequences were found when Beta was phasing out and Delta emerging. So, the sentence is rephrased to;

“Some sequences associated with the Alpha variant were present, but these did not appear to drive a major wave as they did in some other countries, assumably due to the competition from Delta, which is more infectious than Alpha, since Alpha circulated in Malawi when Beta was phasing out and Delta emerging.”

4. The abstract ends with a strong argument about the potential for real-time analysis in Malawi and similar countries. However, it might benefit from a slight rephrasing for impact:

   <u>Proposal:</u> We suggest that real-time analyses should be considered in Malawi and other, where similar computational and data resources are available."

   We rephrased as suggested.

5. The repetition of "Omicron" in "Omicron BA.1; (5) Other Omicron" might be rephrased to avoid redundancy. Consider merging these points, e.g., "Omicron BA.1 and other sublineages."
   We used “other Omicron” to emphasize that there was a fifth major wave dominated by Omicron lineages other than BA.1.

<u>Materials and methods</u>
Considerer rephrasing

1. "Malawi is a landlocked country that is bordered by Mozambique to the East, South and Southwest, Tanzania to the north and northeast and Zambia to the West and Northwest" could be streamlined for clarity.

   <u>Proposal:</u> "Malawi is a landlocked country bordered by Mozambique to the east, south, and southwest; Tanzania to the north and northeast; and Zambia to the west and northwest."
   The sentence was rephrased.

2. "The country is located at latitudes between 9◦ 22' S and 17◦ 03' S and longitudes between 33◦ 40' E and 35◦ 55' E".

   <u>Proposal:</u> "Malawi lies between latitudes 9◦ 22' S and 17◦ 03' S, and longitudes 33◦ 40' E and 35◦ 55' E."
   We accepted the proposal

3. "The country has three regions; North, Central and South; and 28 districts"

Proposal: "Malawi is divided into three regions—North, Central, and South—and comprises 28 districts."
We accepted the proposal.

4. "In a year before the 2018 household census across the country, there were 6.3 all-cause deaths and 32.8 births per 1,000 persons"

   Proposal: "In the year preceding the 2018 household census, the country recorded 6.3 all-cause deaths and 32.8 births per 1,000 persons."
   We accepted the proposal.

5. "Malawi is categorised as a Low-Income country [21] with a fragile health system [22]"

   Proposal: "Malawi is categorized as a Low-Income country [21], with a health system that faces significant challenges, including limited financial and human resources [22]."
   We accepted the proposal.

Results

The overall structure of the "Results" section is logical; however, the readability can be improved by slightly rephrasing some sentences for clarity.

Rephrasing some sentences for clarity

1. "The overall CFR for the whole study period is 3.05% (95% credible interval (2.94, 3.16)%)" is clear but could be more concise.

   Proposal: "The overall CFR for the entire study period was 3.05% (95% CrI: 2.94%–3.16%)."
   We accepted the proposal.

2. "CFR = 4.16%, CrI = (3.93, 4.40)"

   Proposal: "CFR: 4.16% (95% CrI: 3.93%–4.40%)."
   We accepted the proposal.

3. "Delta was more severe (CFR = 4.16%, CrI = (3.93, 4.40)) than the other variants," the word "severe" might be replaced with "lethal" or "had a higher fatality rate," as "severe" might imply clinical severity rather than mortality specifically.
   We accepted the proposal.

4. Case Fatality Rate for SARS-Cov-2 variants"

   Proposal: "Case Fatality Rates for SARS-CoV-2 Variants Observed in Malawi."
   We accepted the proposal.

5. "The full posterior distribution of CFR for the variants is shown in Supplementary Material S1, showing the CFR of Other and Beta overlapping,"

   Proposal: "The full posterior distribution of CFRs, which indicates overlapping CFRs for the 'Other' and Beta variants, is provided in Supplementary Material S1."
   We accepted the proposal.

6. "The posterior distribution of CFR for the variants suggests the same, that Delta has the highest CFR and Omicron has the lowest"

   Proposal: "The posterior distribution of CFRs further confirms that the Delta variant had the highest fatality rate, while the Omicron variant had the lowest."
   We accepted the proposal.

7. "Five peaks are clearly visible in the plot, representing the five waves however, the last peak is very short" could be rephrased for clarity.

   Proposal: "Five distinct peaks are visible in the plot, representing five waves of infection, though the final peak is notably brief."
   We accepted the proposal.

Numerical Data Presentation:

1. Ensure consistent formatting of numerical data throughout the text. For example, "88, 064" should be "88,064" (no space after the comma), and "1, 316" should be "1,316."
   On the tex file there are no spaces, although on the pdf file looks like there are.
2. When mentioning specific numbers like "95 new reported cases every day" and "variance was 41,200," consider adding brief context or comparisons to help readers understand the significance of these figures.
   On lines 159-162, we rephrased to:
   “ 95 people were confirmed infected every day” and:

“variance was 41,200, which implies that there was much variation in the values for the number of daily confirmed cases”.

3. The explanation of the GAM model and its significance is good but could be made clearer. For example: "A Generalized Additive Model (GAM) was fit to the case data using a negative binomial distribution with a log link function. This model accounted for day and day-of-week effects and is visualized in Fig 2C."
   We corrected this.

4. Ensure consistency in how variants are referred to throughout the text. For instance, "Omicron," "Beta," "Delta," and "Other" should always be capitalized and referred to consistently (e.g., "Other" variant instead of "non-VOC").
   We rephrased the sentence as proposed in the next comment.

5. "Malawi’s first variant was non-VOC, which emerged before Alpha (Other)" is somewhat unclear.

   Proposal: "The first variant identified in Malawi was non-VOC, which predated the Alpha variant and is referred to here as 'Other.'"
   We accepted the proposal.

Discussion

Rephrasing some sentences for clarity

1. "The far-reaching consequences of COVID-19 throughout the world as well as individual states motivates the need for extensive research around the pandemic,"

   Proposal: "The global and local impacts of COVID-19 have driven the need for extensive research on the pandemic."
   We accepted the proposal.

2. "It was, therefore, worth understanding the disease dynamics, the evolution of the virus and the burden the pandemic posed on a low-income country like Malawi to aid in the formation of policies and interventions at present or in the future"

   Proposal: "Understanding the disease dynamics, viral evolution, and pandemic burden in a low-income country like Malawi is crucial for informing current and future policy interventions."
   We accepted the proposal.

3. Correct, the sentence contains a typo
   Line 317.
   We corrected the typo

4. “With the use of generalised additive models, te infection’s trend and groethrate were described while the use of IQtree, TreeTime and iTOL allowed the capturing of the times of the most recent common ancestors for the variants as well as the geographical distribution of the virus”.

   This should be corrected to "the infection’s trend and growth rate "
   This was removed as the part was restructured.

Rephrasing some sentences for clarity

5. "Results show that Malawi encountered five waves of SARS-CoV-2 (see Fig 2A), and that each wave was dominated by one variant as the time tree, as Fig 5 illustrates,"

   Proposal: "Results indicate that Malawi experienced five waves of SARS-CoV-2 (see Fig 2A), each dominated by a distinct variant, as illustrated by the time tree in Fig 5."
   This was removed to keep the section concise.

6. "This chronological order agrees with the literature particularly study results of Anscombe (2023)"

   Proposal: "This chronological order is consistent with the findings of Anscombe (2023) [43], who also observed a similar sequence of variant emergence in Malawi."
   We removed this to focus on major findings.

7. "Comparing the waves in terms of cases, the study found that the first wave, Other was the least infectious of all" could be clarified.”

   Proposal: "In comparing the waves by case numbers, the study found that the first wave, dominated by the 'Other' variant, was the least infectious, potentially due to low testing or under-reporting during the early pandemic phase."
   We accepted the proposal.

8. The χ2 test showed that proportions of COVID-19 variants across the regions of Malawi were significantly different implying that the variants posed unequal impact on the regions. Precisely, Omicron was worse in the southern and northern regions compared to the central region. Beta did not affect the southern region much, as Delta did in the north.

   Proposal: The distribution of COVID-19 variants across the regions of Malawi varied significantly, indicating that the impact of the pandemic was not uniform across the country. Specifically, the Omicron variant had a more severe impact in the southern and northern regions compared to the central region. In contrast, the Beta variant had a relatively limited effect on the southern region, while the Delta variant had a more pronounced impact in the northern region.
   We accepted the proposal.

Interpretation of Results:

9. The interpretation of the impact of vaccination and variant infectiousness is clear but could be expanded slightly for depth.

   Proposal: "The reduced infectiousness of the Delta variant, coinciding with the vaccination rollout, suggests a potential impact of vaccination in slowing transmission and establishing herd immunity."
   We accepted the proposal.

10. The statement "Omicron was more aggressive despite that a good proportion of the population had been vaccinated" should be clarified to distinguish between "aggressiveness" in terms of transmission versus severity:

   Proposal: "Omicron was more transmissible despite a substantial portion of the population being vaccinated, though it resulted in fewer deaths compared to Beta and Delta."
   We accepted the proposal.

Conclusions

The conclusion effectively recaps the main findings, but it would be better with a more organized synthesis. You could consider grouping related findings together. For example:
1) Summarize the variant distribution 2) Discuss the impact on case fatality rates 3) Conclude with recommendations for future focus areas.
For example:

Proposal: "The study identified five major waves of SARS-CoV-2 in Malawi, each dominated by different variants, with Delta and Beta being the most prevalent. While the overall case fatality rate was lower than in high-income countries, it was higher than in other low-income countries. The study also highlighted the critical periods of increased transmission and emphasized the need for targeted interventions during these times."
We added this to the conclusion

Proposal: "To mitigate future outbreaks, it is crucial to prioritize resource allocation to the northern and southern regions, which were most affected by Omicron. Additionally, ongoing surveillance and rapid response strategies should be emphasized, particularly during periods of high transmission."
We added this to the conclusion.

Proposal: "Further research is needed to explore the role of pre-existing immunity, genetic factors, and the impact of early preventive measures on disease outcomes in Malawi."
We added this to the conclusion.

Proposal: "Given the rapid genetic evolution of SARS-CoV-2, continued vigilance and adaptability in public health strategies are essential to manage future variants and pandemics."
We added this to the conclusion.

Complete the reference.
Now: of Malawi PHI. COVID-19 Surveillance Dashboard; 2022.
Correct: The Public Health Institute of Malawi PHI. COVID-19 Surveillance Dashboard; 2022
This was corrected, see reference number 21.
